# *Wolbachia* lipoproteins: abundance, localisation and serology of *Wolbachia* peptidoglycan associated lipoprotein and the Type IV Secretion System component, VirB6 from *Brugia malayi* and *Aedes albopictus*

**DOI:** 10.1186/s13071-014-0462-1

**Published:** 2014-10-06

**Authors:** Denis Voronin, Ana F Guimarães, Gemma R Molyneux, Kelly L Johnston, Louise Ford, Mark J Taylor

**Affiliations:** Department of Parasitology, Liverpool School of Tropical Medicine, Pembroke Place, Liverpool, L3 5QA UK

**Keywords:** *Wolbachia*, Lipoprotein, Peptidoglycan associated lipoprotein, Type IV Secretion System VirB6, *Brugia malayi*, *Aedes albopictus*

## Abstract

**Background:**

Lipoproteins are the major agonists of *Wolbachia*-dependent inflammatory pathogenesis in filariasis and a validated target for drug discovery. Here we characterise the abundance, localisation and serology of the *Wolbachia* lipoproteins: *Wolbachia* peptidoglycan associated lipoprotein and the Type IV Secretion System component, VirB6.

**Methods:**

We used proteomics to confirm lipoprotein presence and relative abundance; fractionation, immunoblotting and confocal and electron immuno-microscopy for localisation and ELISA for serological analysis.

**Results:**

Proteomic analysis of *Brugia malayi* adult female protein extracts confirmed the presence of two lipoproteins, previously predicted through bioinformatics: *Wolbachia* peptidoglycan associated lipoprotein (wBmPAL) and the Type IV Secretion System component, VirB6 (wBmVirB6). wBmPAL was among the most abundant *Wolbachia* proteins present in an extract of adult female worms with wBmVirB6 only detected at a much lower abundance. This differential abundance was reflected in the immunogold-labelling, which showed wBmPAL localised at numerous sites within the bacterial membranes, whereas wBmVirB6 was present as a single cluster on each bacterial cell and also located within the bacterial membranes. Immunoblotting of fractionated extracts confirmed the localisation of wBmPAL to membranes and its absence from cytosolic fractions of C6/36 mosquito cells infected with *w*AlbB. In whole worm mounts, antibody labelling of both lipoproteins were associated with *Wolbachia.* Serological analysis showed that both proteins were immunogenic and raised antibody responses in the majority of individuals infected with *Wuchereria bancrofti*.

**Conclusions:**

Two *Wolbachia* lipoproteins, wBmPAL and wBmVirB6, are present in extracts of *Brugia malayi* with wBmPAL among the most abundant of *Wolbachia* proteins. Both lipoproteins localised to bacterial membranes with wBmVirB6 present as a single cluster suggesting a single Type IV Secretory System on each *Wolbachia* cell.

## Background

*Wolbachia* are one of the most abundant intracellular symbiotic bacteria in nature. They are most common in terrestrial arthropods, with up to 40% of species infected, and are also found in a sub-group of filarial nematodes, where they have evolved a mutualistic association [1,2]. In filarial nematodes *Wolbachia* are essential for normal larval growth and development, embryogenesis and the survival of adult worms. Loss of *Wolbachia* induces extensive apoptosis of germline and somatic cells, presumably due to the lack of provision of an essential nutrient or metabolite required to prevent apoptosis of these cells and tissues during periods of high metabolic demand [3]. This mutualistic association has been exploited as a target for antibiotic therapy, which can cure patients infected with *Wuchereria bancrofti* and *Onchocerca volvulus*, providing an alternative treatment and control strategy [1,4,5].

In addition to their essential role in the biology of filarial nematodes, *Wolbachia* are a major driver of the inflammatory pathogenesis of filarial disease [6]. Previous studies have determined that the pro-inflammatory capacity of *Brugia malayi* and *Onchocerca volvulus* is dependent on the presence of *Wolbachia* and the molecular ligands responsible have been characterised as lipoproteins [7]. The induction of innate and adaptive inflammatory responses is dependent on recognition by Toll-like receptors 2 and 6 (TLR2/6) [7,8], pattern recognition receptors for di-acylated bacterial lipoproteins. Analysis of the *Wolbachia B. malayi* (*w*Bm) genome revealed the absence of the gene for the apolipoprotein N-acyltransferase (Lnt), which converts di- to tri- acylated bacterial lipoprotein in gram-negative bacteria, suggesting lipoproteins in *Wolbachia* are di-acylated accounting for their recognition by TLR2/6 [7].

Bioinformatic analysis of *w*Bm genome using three distinct databases consistently identified only two lipoproteins [7]: 1) *Wolbachia* peptidoglycan-associated lipoprotein (wBmPAL) and 2) *Wolbachia* VirB6 (wBmVirB6), which is a core component of the bacterial Type IV secretion system (TIVSS) [9]. Furthermore, a chemically synthesised *Wolbachia* PAL di-acylated lipopeptide replicated the inflammatory activity of whole *Wolbachia* and soluble parasite extracts [7]. Here, we have further characterised these two *Wolbachia* lipoproteins to determine their relative abundance, structural localisation in the filarial nematode *Brugia malayi* (naturally infected with *w*Bm) and *Ae. albopictus* cell line (C6/36 infected with *w*AlbB) and serology in people infected with *W. bancrofti*.

## Methods

### Parasite material, mosquito cell line culture and patient serum

Adult *B. malayi* cultivated in the peritoneal cavity of jirds (*Meriones unguiculatus*) were derived from TRS Laboratories (Athens, Georgia, USA) and maintained at the Liverpool School of Tropical Medicine. Infected animals received tetracycline at 2.5 mg/ml in drinking water for a period of 6 weeks. Control infected jirds were maintained in a similar fashion but without the tetracycline. Two weeks after the end of the treatment, worms were collected from the peritoneal cavities using preheated (37°C) culture medium RPMI-1640 (GIBCO). All procedures involving the use of laboratory animals were approved by the Ethics and Animal Care Committees of the University of Liverpool and Liverpool School of Tropical Medicine, and were carried out according to the Animals (Scientific Procedures) Act (UK Home Office).

*Ae. albopictus* mosquito cell lines C6/36 (*w*AlbB) infected with *Wolbachia* strain *w*AlbB and non-infected C6/36 (NI) are maintained in culture at the Liverpool School of Tropical Medicine, Liverpool, UK [10]. Cells were cultivated routinely in 25 cm^2^ plastic culture flasks at 26°C in 5 ml of Leibovitz-15 medium containing 2 mM L-glutamine (Life Technologies), 1% non-essential amino acids, 2% tryptose phosphate broth, 50 U/ml penicillin, 50 μg/ml streptomycin (Sigma) and 5% heat-inactivated FBS (Perbio). Cells were sub-cultured into new flasks every 4–5 days.

Serum samples from *W. bancrofti* infected individuals were collected as part of a clinical trial in Tanzania. The samples used for serology were collected prior to any drug treatment. The study was approved by the ethics research committees of the National Institute for Medical Research, Dar es Salaam, Tanzania, and the Liverpool School of Tropical Medicine, Liverpool, UK. Written and oral informed consent was obtained from all participants [11].

### Fractionation of mosquito cells

Mosquito cells were homogenised in PBS buffer by vortexing with 3 mm borosilicate glass beads. Homogenates were then centrifuged for 5 min at 300 × g to eliminate cell debris. Supernatants containing intracellular components were pelleted by centrifugation at 8,150 × g for 5 min, and re-suspended in 330 μl PBS. Cellular suspensions were sonicated on ice for a period of 1 min (1 s run/2 s pause) at 1,800 J (cumulative value). The supernatants were pooled and cellular complexes were removed by centrifugation at 8,150 × g for 10 min. The supernatant was overlaid on 1 ml of sucrose solution and cellular membranes were pelleted from the supernatants by ultracentrifugation in TLA-100 (Beckman) at 430,000 × g for 1 h at 4°C. The proteins were precipitated from the supernatant to obtain the cytosolic fraction and from the pellet for the membrane fraction using acetone. Samples were stored at –80°C until use.

### Western blot

Antibodies to wBmPAL were generated as described previously [9]. Anti-VirB6 antibodies were produced by New England Biolabs, (USA) using a similar procedure. *B. malayi* worms were collected from antibiotic-treated and untreated jirds, washed three times in PBS and proteins were extracted using sonication. Fractions of C6/36 cells and intact cell pellets were collected in the separate tubes and proteins were extracted using sonication. The protein concentration was estimated by bicinchoninic acid assay (Invitrogen) following the manufacturer’s instructions. Protein extracts of worms were mixed with LDS sample buffer (NuPAGE; Invitrogen), boiled, and run with a 12% PAGE gel. Protein was transferred to nitrocellulose membranes and used in the Western blot as previously described [12].

### Microscopy

*B. malayi* adult females were fixed using 4% formaldehyde in PBS with 0.05% Triton-X100 (PBST) for 20 min for confocal microscopy analysis of the localisation of the proteins. During fixation, worms were cut to improve diffusion of the fixative and antibodies. Samples were then washed three times in PBST and treated with RNase A (100 μg/ml) at 36°C for 1 h. Then samples were washed in PBS and blocked with 5% BSA for 15 min and incubated overnight at 4°C with anti-VirB6 antibody diluted 1:200. Secondary antibody labelled with FITC was used at 1:500. After incubation with antibodies samples were co-stained with propidium iodide for 20 min to visualise DNA (host nuclei and *Wolbachia*) and were viewed with an LSM 5 Pascal confocal microscope (Zeiss).

For immuno-TEM, worms and C6/36 cells were fixed by 4% paraformaldehyde dissolved in PBS for 4 h at 4°C. During fixation, worms were cut. After fixation, samples were washed in PBS (three times on ice). Fixed mosquito cells were embedded into low-melting agarose. All samples were then dehydrated in a series of ethanol concentrations (50–100%) on ice. Dehydrated samples were embedded in Lowicryl Gold plastic resin. Ultrathin sections were blocked using 5% BSA and incubated with primary antibodies diluted 1:100 in 1% BSA overnight at 4°C. The next day, sections were washed three times in PBS and incubated with secondary antibody labelled with 15 nm gold particles. Sections were washed and contrasted using uranyl acetate (1%) and lead citrate and analysed under the Tecnai G2 Spirit BioTWIN TEM (the TEM unit, University of Liverpool, UK). For TIVSS analysis serial sections were taken every 70-90 nm to cover the entire bacterial cell of non-dividing *Wolbachia.*

### High resolution mass spectrometric analysis of total parasite protein

#### Proteomics

For proteomic analysis, a total of 18 adult female *B. malayi* were collected by washing out the peritoneal cavity of Mongolian jirds (*Meriones unguiculatus*) with warm RPMI 1640 media not containing FBS. Worms were processed by sonication (in 25 mM ammonium bicarbonate) at 70% amplitude for 30 s with 30 s rest on ice (a total of three cycles). Proteins were treated with the surfactant 0.1% (v/v) RapiGestTM (Waters) at 80°C for 10 min followed by reduction with diothiothreitol (DTT) at a final concentration of 3 mM (60°C for 10 min) and alkylation with iodoacetamide (IAA) at a final concentration of 50 mM (room temp, in the dark, 1 h). The enzyme trypsin (sequencing grade, Promega) was added at an enzyme: substrate ratio of 1:50 and incubated overnight at 37°C. The surfactant was inactivated the following day by treatment with 0.1% trifluoroacetic acid (TFA) (37°C for 1 h), peptides were recovered following centrifugation at 13,0000 g. A proportion of unfractionated peptide sample was retained for mass spectrometric analysis. A total of 150 μg of digested protein material was subjected to extensive fractionation over the pH range 3-10 using the Agilent 3100 OFFGEL fractionator system as per manufacturer’s instructions.

#### Reversed-phase liquid chromatography- tandem mass spectrometry (RPLC-MSMS)

Both the unfractionated and fractionated peptide samples were separated by RPLC using a DIONEX UltiMateTM 3000LC chromatography system and MSMS analysis performed on an LTQ Orbitrap Velos using Xcalibur software v2.1 (Thermo Scientific, UK). Peptides (10 μl = ~500 ng) were injected onto the analytical column (Dionex Acclaim® PepMap RSLC C18, 2 μm, 100 Å, 75 μm i.d. × 15 cm, nanoViper.), which was maintained at 35°C and at a nanoflow rate of 0.3 μlmin^-1^. Peptides were separated over linear chromatographic gradients composed of buffer A (2.5% acetonitrile [ACN]: 0.1% formic acid [FA]) and buffer B (90% ACN: 0.1% FA). Two gradients, 60 (3-50% buffer B in 40 min) and 180 min (3-60% buffer B in 140 min), were employed for analysis. Full scan MS spectra were acquired over the *m/z* range of 350-2000 in positive polarity mode by the Orbitrap at a resolution of 30,000. A data-dependent Top20 collision induced dissociation (CID) data acquisition method was used. The ion-trap operated with CID MSMS on the 20 most intense ions (above the minimum MS signal threshold of 500 counts).

#### Protein identification

All MSMS data generated was searched against a customised database for *Wolbachia* (*wBm*) and *B. malayi* (concatenated from .fasta files obtained from UniProtKB www.uniprot.org, downloaded on 14/01/2013) using the search engine MASCOT and Proteome Discoverer software v1.2 (Thermo Scientific, UK). Search parameters included a precursor mass tolerance of 10 ppm and fragment ion tolerance 0.8 Da with one tryptic missed cleavage permitted. Carbamidomethyl (C) was set as a static modification with oxidation of methionine (M), deamidation (N, Q) and phospho (ST) (Y) set as dynamic modifications. A decoy database was searched and relaxed peptide confidence filters applied to the dataset (ion scores *p <* 0.05/FDR 5%).

### IgG antibody ELISA

Flat-bottomed 96-well ELISA plates (Immulon®4HBX ultra high binding polystyrene microtiter plates) were coated overnight at 4°C with 50 μl/well of 5 μg/ml of *Wolbachia* (PAL and VirB6) recombinant proteins diluted in carbonate-bicarbonate buffer, pH 9.6. Plates were covered with plastic film to prevent drying. The following day, plates were blocked with 5% skimmed milk powder in PBST (0.05% Tween 20 in PBS, pH 7.4), covered as previously and incubated for 1 h at 37°C. Plates were then inverted and washed with PBST. Human serum samples (50 μl/well), at a final dilution of 1:200 in blocking buffer, were added to the plates, which were then covered and incubated for 2 h at room temperature (RT). Plates were inverted and washed. 50 μl/well of primary antibody murine anti-human IgG (1 mg/ml; Skybio, UK) diluted 1:1000 in PBST were added to separate plates, which were then covered and incubated for 1 h at RT. After inversion and washing, 75 μl/well of secondary antibody anti-murine rabbit IgG conjugated with horseradish peroxidase (HRP) at 1 mg/ml (Invitrogen) diluted 1:2000 in PBT was added to each plate. Plates were covered and incubated for 1 h at RT. After inversion and washing, 100 μl/well of TMB substrate and chromogen solution (Sigma) was added to each plate and these were incubated at RT in the dark until the reaction was stopped after 30 min by adding 50 μl/well of 1 M H_2_SO_4_. The values of optical density were read at 450 nm wavelength. Cut-off values were then defined as three times the standard deviation plus the mean of the European control sera for each antigen tested. The Mann Whitney test (used to compare the differences in median antibody levels between the different clinical groups) was carried out using GraphPad Prism (5.0a Mac OS X).

## Results

### Identification of *Wolbachia* lipoproteins in *Brugia malayi* adult female worms by proteomic and mass spectrometric analysis

**Figure 1:**
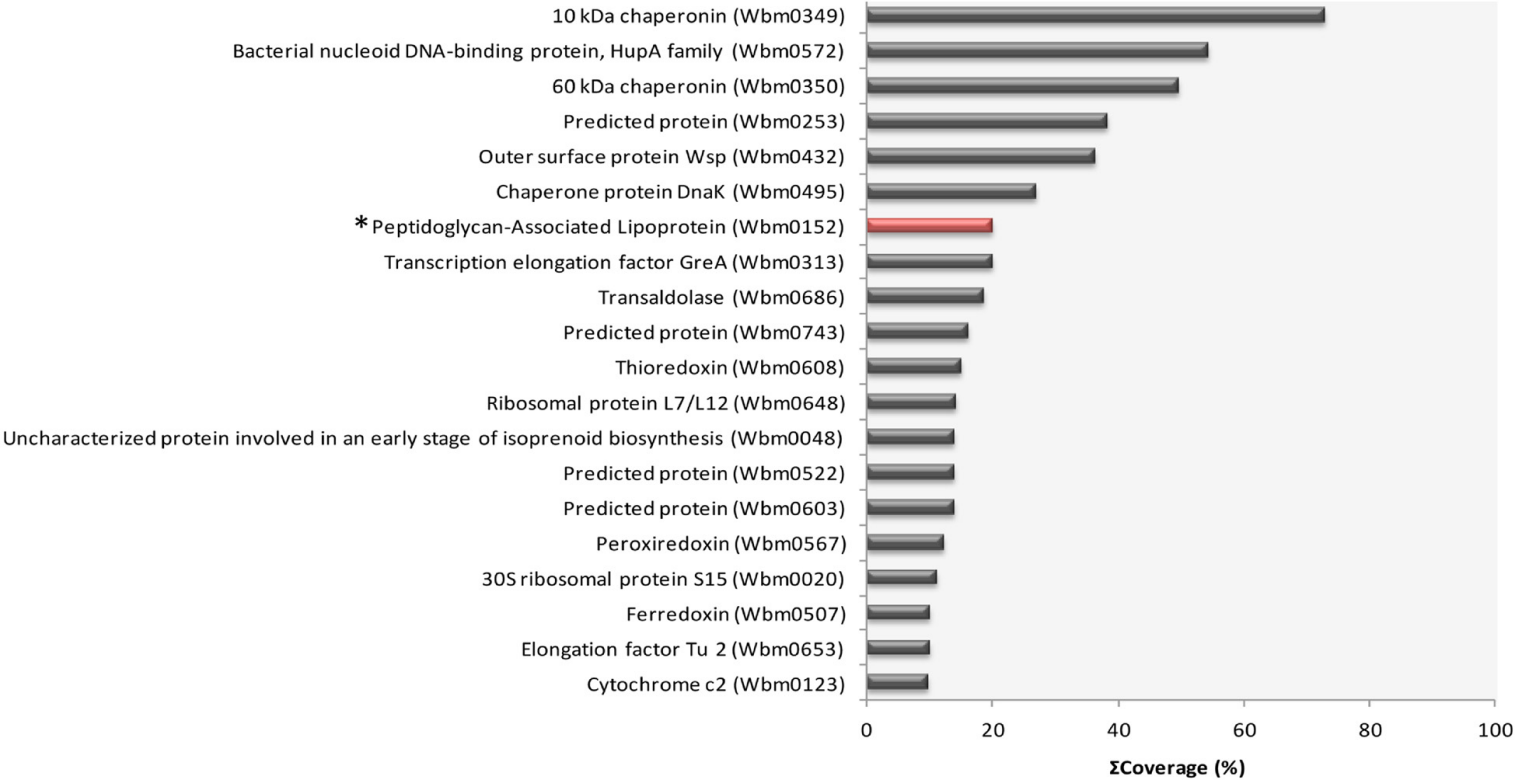
**Analysis of the** ***Wolbachia*** **proteome of adult female** ***Brugia malayi.*** *Wolbachia* proteins identified by proteomic and high resolution mass spectrometric analysis were filtered to report only high/medium confidence peptides then ranked according to protein sequence coverage observed (top 20 shown). The presence of predicted lipoprotein, Peptidoglycan-Associated Lipoprotein (Wbm0152), was confirmed by spectral matching of MSMS data against a customised protein sequence database to two unique tryptic peptides (MASCOT ion scores *p <* 0.05/FDR 5%) constituting 20.13% total protein sequence coverage; it is positioned within the top 10 proteins in this ranked list (*).

Using a global proteomics approach coupled with high resolution mass spectrometric analysis of protein extract, we were able to confirm the presence of two predicted *Wolbachia* lipoproteins in adult female *B. malayi*. We have detected outer membrane protein, PAL (Wbm0152) and TIVSS, VirB6 components (Wbm0794) with high confidence, following manual validation of MSMS data. *Wolbachia* proteins have been ranked according to protein sequence coverage observed and the top 20 proteins from this ranked list are shown in Figure 1. The highest degree of lipoprotein sequence coverage was observed for PAL (~20%) with VirB6 (Wbm0794) confirmation based upon single peptide identification, with reduced (<1%) protein sequence coverage. We were also able to successfully differentiate between two paralogues of the TIVSS components, VirB6 (Wbm0793 and Wbm0794) by unique peptide identification. Both paralogues were identified with similar reduced (<1%) protein sequence coverage. Paralogue Wbm0793 was not predicted as a lipoprotein by any of the bioinformatic tools used in this study (Database of Lipoproteins, LIPO, LipoP).

### Localisation of *Wolbachia* Peptidoglycan-Associated Lipoprotein (wPAL) in *Aedes albopictus* mosquito cells and *Brugia malayi* adult worms

**Figure 2:**
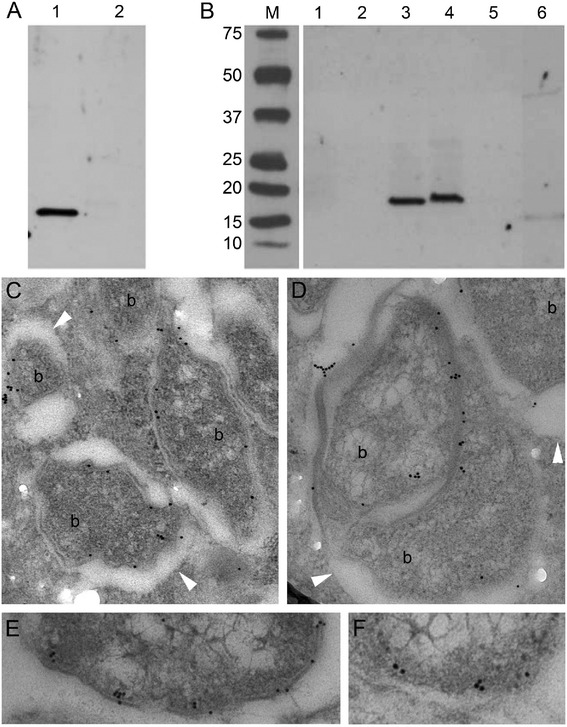
**Localisation of PAL on** ***Wolbachia*** **membranes. A**, **B** – Western immunoblot detection of wPAL protein in *B. malayi* **(A)** and C6/36 mosquito cell line fractional **(B)** samples. **A**. Protein samples of *B. malayi* infected *w*Bm (1) and *B. malayi* after six weeks tetracycline treatment (2). **B**. Protein samples of infected and non-infected C6/36 cells cultivated *in vitro*: 1 – total protein extract of non-infected cells, 2 – membrane fraction of non-infected cells, 3 – total protein extract of cells infected with *w*AlbB, 4 – membrane fraction of infected cells, 5, 6 – cytoplasmic fractions of non-infected and infected cells, respectively. M – marker, kDa. **C**-**F** – Immuno-transmission electron microphotographs showed localisation of PAL on the bacterial membrane in cytoplasm of adult *B. malayi* infected with wBm. b – bacteria. Arrow head indicates membrane of vacuole containing *Wolbachia*.

We used an affinity purified polyclonal rabbit anti-wBmPAL antibody to perform western blot analysis of the proteins extracted from the *Wolbachia-*infected mosquito cell line C6/36 (*w*AlbB) and *B. malayi* (*w*Bm) females. As demonstrated previously, protein extracts from *B. malayi* females showed a single band at 17 kDa, which was lost following depletion of *Wolbachia* with tetracycline (Figure 2A, [9]). *Aedes albopictus* mosquito cells (C6/36 line) were crushed to extract total proteins, cytosolic protein fractions, and membrane protein fractions for western blot analysis (Figure 2B). The crude extract of the cells infected with *w*AlbB and the membrane fraction from C6/36 (*w*AlbB) showed positive signals for PAL, whereas the cytosolic fraction from C6/36 (*w*AlbB) showed no reactivity. A slight difference in migration of PAL in lanes 3 and 4 may relate to differences in preparation of the extracts with enriched membrane proteins running slightly slower in the gel. Non-infected C6/36 (NI) cells were used as a negative control and the protein extracts from all fractions showed no wPAL reactivity (Figure 2B).

Next we investigated the ultrastructural localisation of wBmPAL in *B. malayi* females using transmission electron microscopy. We observed a specific localisation of anti-wBmPAL antibodies to the membranes of *Wolbachia* (Figure 2C, D)*.* Immunogold particles were not observed on the membranes of vacuoles containing *Wolbachia*. Statistical analysis of the distribution of immunogold particles on the sections of *B. malayi* lateral cord showed 86% of all counted particles located on the bacterial membrane and only 14% in the cytoplasm of the host cell (Table 1). The low number of immunogold particles that were randomly distributed in the cytoplasm represented the level of background staining and this level was similar in all the control experiments (tetracycline treated *B. malayi* and *Wolbachia*-free *Acanthocheilonema viteae*, Table 1).

**Table 1:** **Statistical analysis of gold-dot distribution on the TEM sections of** ***B. malayi*** **and** ***A. viteae***

**Immunogold distribution on the sections stained with anti-PAL antibody**		
	**Bacteria (mean ± SD, %)**	**Host cytoplasm (mean ± SD, %)**
BM	86.6 ± 18.5*, ^, “	13.4 ± 11.7
BM-TET	0	11.7 ± 5.0
AV	0	5.2 ± 3.0

* - Statistical analysis of means of immunogold particles located on bacterial material and particles located on host cytoplasm in *B. malayi* (BM), p-value < 0.01.

^ - Statistical analysis of means of immunogold particles located on bacterial material in *B. malayi* (BM) and particles located on host cytoplasm of tetracycline treated *Brugia malayi* (BM-TET) p-value < 0.01.

“ - Statistical analysis of means of immunogold particles located on bacterial material in *B. malayi* (BM) and particles located on host cytoplasm of *A. viteae* (AV) p-value < 0.01.

### Localisation of *Wolbachia* VirB6, a core component of Type IV Secretion System in *Aedes albopictus* mosquito cells and *Brugia malayi* adult worms

**Figure 3:**
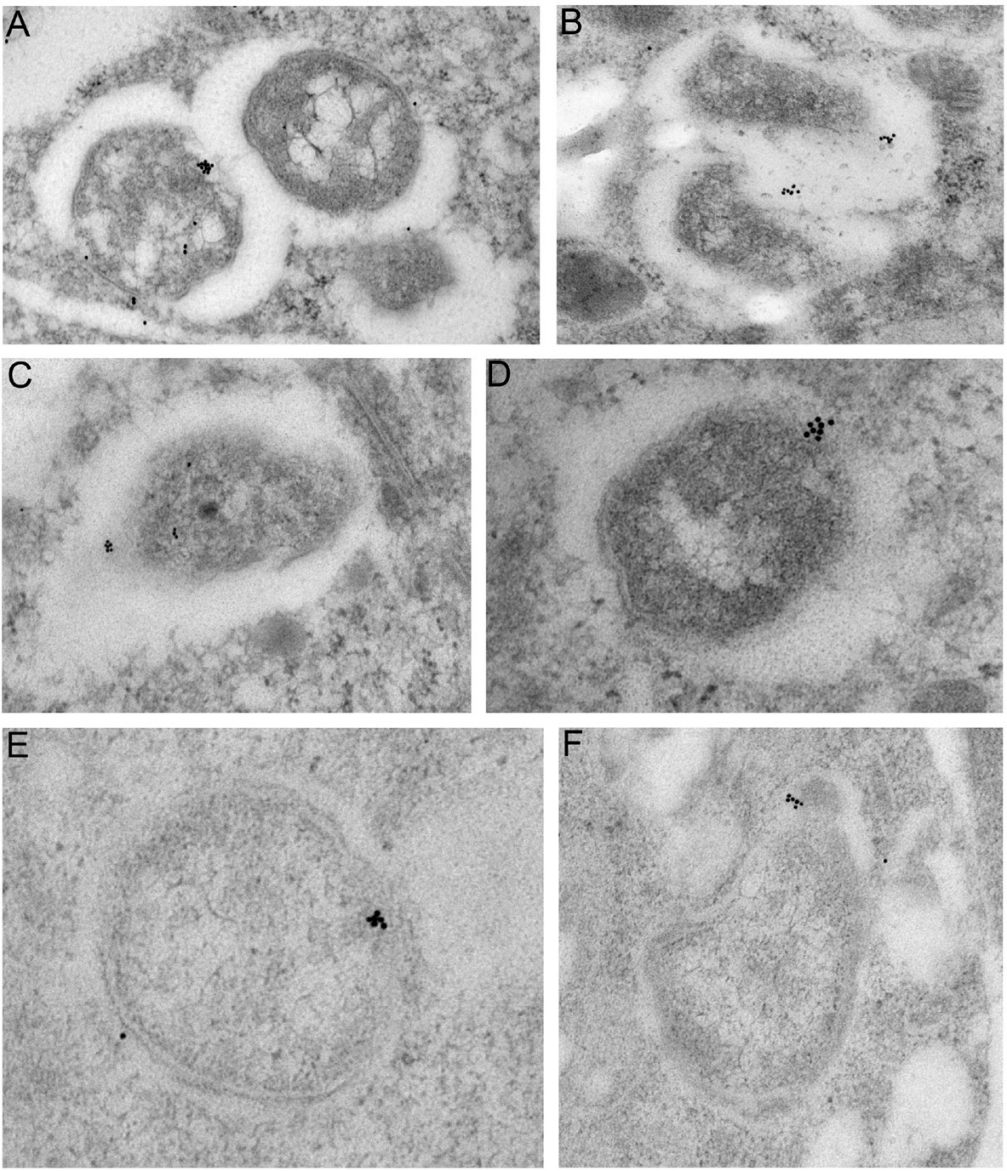
**Ultrastructural distribution of** ***Wolbachia*** **VirB6 in lateral cord of** ***Brugia malayi.*** Immuno-transmission electron microphotographs showing distribution of *Wolbachia* VirB6 in lateral cord of *Brugia malayi* and a mosquito cell line. **A**, **B** – localisation of VirB6 in a bacterium-bacterium connection in cytoplasm of lateral cord of adult *B. malayi*, **C**, **D** – a single complex of VirB6 detected on the bacterial pole in developing microfilaria, **E**, **F** – VirB6 on *w*AlbB bacterial membrane in cytoplasm of mosquito cells (C6/36 cell line).

Antibodies raised to wBmVirB6, were unsuitable for western blot analysis due to non-specific binding. Immunogold ultrastructural localisation of VirB6 in host cells was determined using immuno-TEM analysis of *B. malayi* females and C6/36 (*w*AlbB) cells. *Wolbachia* VirB6 protein localised as a single discreet cluster(s) on the *Wolbachia* membranes (Figure 3A, B)*.* In addition, occasional immunogold labelling was also observed localised within the bacterial matrix (Figure 3C, D), which could represent a precursor of the protein synthesised in bacteria. This pattern of protein distribution was similar in somatic cells and developing embryos of *B. malayi* (Figure 3A-D). Serial sections of bacteria determined that the VirB6 protein clusters as a single complex established in bacterial membranes and on some sections near the pole of bacteria. In some cases *Wolbachia* had two clusters, which were located on the opposite poles prior to division.

### Confocal microscopy

**Figure 4:**
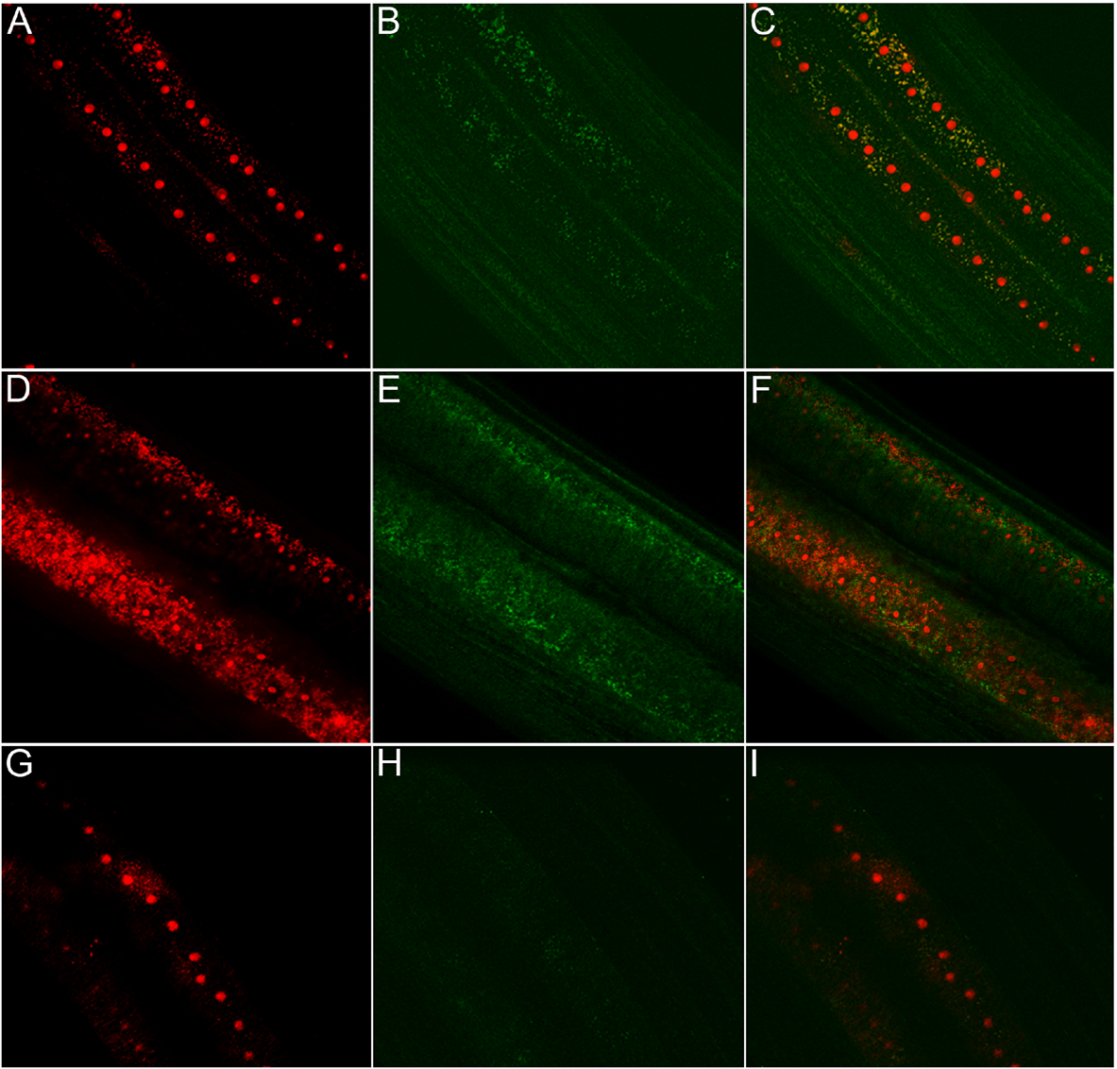
**Visualisation of PAL and VirB6 in whole worm mounts of** ***B. malayi*** **. A**, **B**, *Wolbachia* (small red foci, bacteria DNA stained by propidium iodide, large red spot, nematode nuclei, see Methods) and PAL (green, secondary antibody labeled by FITC) in lateral cord of filarial nematode, **C**, merged **A** and **B**. **D**, **E**, VirB6 (green) and *Wolbachia* (small red foci) in *B. malayi* (wBm). **F**, merged **D** and **E**. **G**, **H**, negative control – *Wolbachia*-free *B. malayi* treated with antibiotic (lack of PAL reactivity shown as representation of both antibodies). **I**, merged **G** and **H**.

To determine whether the protein localisation was associated with *Wolbachia* location in whole organisms or cells, we used whole mount confocal microscopy. The location of antibodies to both wBmPAL and wBmVirB6 were associated with *Wolbachia* (stained by propidium iodide, Figure 4A-F) in the hypodermal cord cells of *B. malayi*. Antibody reactivity was absent from tetracycline treated *B. malayi* (Figure 4G-I)*.*

### Serology

**Figure 5:**
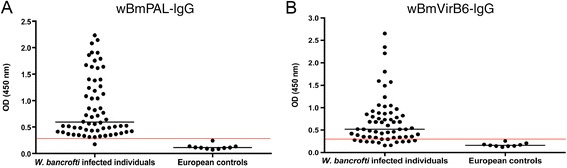
**IgG antibody responses against** ***Wolbachia*** **lipoproteins in patients infected with** ***Wuchereria bancrofti*** **.** IgG reactivity against wBmPAL **(A)** and virB6 **(B)**. Infected groups both show significantly elevated levels of IgG to both lipoproteins (*P* < 0.05) compared with European controls. The red line represents the cutoff value (mean + 3 X SD).

Next, we investigated the human IgG response to the two lipoproteins in people infected with *W. bancrofti* from Tanzania. Both *Wolbachia* lipoproteins showed high levels and frequency of antibody reactivity in infected individuals (Figure 5) with 98.4% and 82.3% of patients demonstrating IgG responses to wBmPAL and wBmVirB6 respectively.

## Discussion

Here we show that two *Wolbachia* lipoproteins, PAL and VirB6 are located on the membranes of *Wolbachia* (*w*Bm and *w*AlbB). Proteomic analysis of *B. malayi* adult female protein extracts confirmed the presence of two lipoproteins, previously predicted through bioinformatics; *Wolbachia* peptidoglycan associated lipoprotein (wBmPAL) and the Type IV Secretion System component, VirB6 (wBmVirB6). Using protein sequence coverage as an estimate of relative abundance, we showed that wBmPAL was among the most abundant *Wolbachia* proteins present in an extract of adult female worms with wBmVirB6 only detected at a much lower abundance. This high abundance of PAL has been previously reported in adults and microfilariae of *B. malayi* [13] and *O. ochengi* [14] in which PAL consistently appears in the top 10 abundant *Wolbachia* proteins. This differential abundance was reflected in the immunogold-labelling, which showed wBmPAL localised at numerous sites within the bacterial membranes, whereas wBmVirB6 was present as a single cluster on each bacterial cell and also located within the bacterial membranes. Immunoblotting of fractionated extracts confirmed the localisation of PAL to membranes and its absence from cytosolic fractions of C6/36 mosquito cells infected with the *w*AlbB strain.

Our observations are consistent with previous studies, which localise PAL to the membranes of other gram-negative bacteria such as *E. coli* [15]. The typical cell envelope structure of gram-negative bacteria consists of three layers: outer and inner membranes with a thin layer of peptidoglycan (PG) located between the membranes (the periplasmic space). The N-terminus of PAL containing the lipid moiety is anchored to the inner face of the outer membrane, with the C-terminus binding to PG via a pocket for m-DAP residues crosslinking the outer membrane to PG [16]. However, *Wolbachia* does not contain the entire biosynthesis pathway for PG [17], and electron microscopy analysis of the *Wolbachia* cell wall confirms the absence of a PG layer [18,19]. Some components of the biosynthesis of PG are still intact, for example, lipid II, which, in addition to its role in the cell envelope architecture, has a role in cell division [11]. In gram-negative bacteria PAL anchors to the outer membrane but is located in the periplasmic space as part of the Tol–PAL complex [20,21] and evidence for the existence of the periplasmic component of the Tol transport systems (Wbm5057) is found in *w*Bm. This system plays important roles in the transport of molecules through the outer membrane of bacteria, in the regulation of the expansion of membranes and in bacterial sensitivity to antibiotics [21]. The complex is also involved in the protection of bacteria against the penetration of phage particles into the cell and in the formation of cell envelopes in daughter cells [22,23].

The lipoprotein, VirB6, was also localised to the membranes of *Wolbachia* in both *w*AlbB and *w*Bm. In contrast to PAL, which was widely distributed throughout the membranes, VirB6 was localised as a discreet single complex on the bacterial cell. Through the use of serial sectioning at intervals of 70-90 nm and covering the entire bacterial cell of non-dividing *Wolbachia,* we observed that *Wolbachia* has a single TIVSS complex for each cell. This is also observed for the TIVSS complex in *Agrobacterium* [24–26]. During bacterial division, the components of the secretion system migrate onto the pole of *Agrobacterium* [27,28]. On the micrographs of the sections for both insect cells and filarial nematodes, VirB6 was found on the membranes of the *Wolbachia* forming a single complex close to the pole of the bacterium. In detailed analysis of immuno-TEM sections, the complex of VirB6 proteins on the bacterial membrane were often observed in close association with the vacuolar membrane.

The bacterial VirB6 protein is a core component of TIVSS and plays important roles in the transfer of DNA-protein complexes to induce bacterial adaptation processes, to deliver pathogens into host cytoplasm, and to establish bacterial effects on host biology [28–31]. In gram-negative bacteria, TIVSS connects two bacterial membranes and forms a canal-like structure [30]. Previous analysis of the *Wolbachia* VirB6 gene in genomes of different *Wolbachia* strains showed several predicted isoforms [32]. In this study we identified two isoforms from proteomic analysis, only one of which (Wbm0794) was predicted as a lipoprotein by bioinformatics. Computational analysis of the structure of VirB6 protein revealed that the 5-7 transmembrane domains were located on the inner bacterial membrane [30]. The TIVSS is likely to function as a major pathway for the secretion of bacterial products, which are involved in the *Wolbachia*-host symbiosis and a number of *Wolbachia* proteins, including both lipoproteins, can be detected in the parasite secretome [33]. Rickettsial bacteria related to *Wolbachia* such as *Anaplasma* and *Ehrlichia* use TIVSS for successful infection of mammalian cells [29].

The lipoprotein biosynthesis pathway of *Wolbachia* has been investigated as a potential chemotherapeutic target for filariasis [34]. Treatment of *Wolbachia*-infected mosquito cells with the signal peptidase II (LspA) inhibitor, globomycin, led to a dose-dependent reduction in *Wolbachia* load preceded by an inhibition of lipoprotein processing. Treatment of *B. malayi* with globomycin *in vitro* resulted in significant reductions in motility and viability validating this as a target for drug discovery. Similarly, treatment of the related bacteria *Ehrlichia chaffeensis* with globomycin, prevented infection of HL-60 *in vitro*, which was associated with inhibition of lipoprotein processing [35]. PAL specifically has been implicated in this infection process, as antibodies to PAL (OmpA) bind to *E. chaffeensis* and prevent infection of cells *in vitro* [36]. Furthermore PAL of *Helicobacter pylori*, known as OprL, has been defined as a critical drug target *in silico* [37] suggesting that targeting PAL and/or lipoprotein biosynthesis could deliver broad spectrum antibiotic activity.

*Wolbachia* lipoproteins are the agonists of inflammatory pathogenesis of filarial disease through activation of innate and adaptive immunity via recognition by TLR2/6 [6,7]. Bacterial lipoproteins play an important role in the pathogenesis of several bacterial infections including *Yersinia pestis, Pseudomonas aeruginosa, Salmonella enterica, Nesseiria meningitidis, Borrelia burdorferi* and *Mycobacterium tuberculosis* [38]. In sepsis, *E. coli* PAL is a potent TLR2 agonist, which contributes to inflammation, cardiac dysfunction, endothelial activation, coagulopathy, and vascular leakage [39–42]. In filariasis *Wolbachia* PAL recruits neutrophils, macrophages and other innate immune cells, which accumulate around living *Onchocerca* adults worms in onchocermata [43,44] and in response to dying microfilariae in the cornea [45,46], which stimulates their activation and the production of an array of pro-inflammatory cytokines and mediators [6,9]. *Wolbachia* lipoproteins also drive adaptive Th1 immunity through activation of dendritic cells [9]. In view of the relative abundance of PAL compared to VirB6, it seems likely that PAL is the major driver of *Wolbachia*-mediated inflammatory immunity. Exposure of the host to lipoproteins will occur on the release of *Wolbachia* on the death of the parasite or through their presence in the parasite secretome [33]. The high level and frequency of antibodies against these proteins in patients infected with *W. bancrofti* is supportive of their inherent immunogenicity and exposure to the adaptive immune system.

## Conclusion

Two *Wolbachia* lipoproteins, wBmPAL and wBmVirB6, are present in extracts of *Brugia malayi* with wBmPAL among the most abundant of *Wolbachia* proteins. Both lipoproteins localised to bacterial membranes with wBmVirB6 present as a single cluster suggesting a single Type IV Secretory System on each *Wolbachia* cell. Serological analysis showed that both proteins were immunogenic and raised antibody responses in the majority of individuals infected with *Wuchereria bancrofti*.
